# A qualitative study on hope in iranian end stage renal disease patients undergoing hemodialysis

**DOI:** 10.1186/s12882-023-03336-6

**Published:** 2023-09-22

**Authors:** Masoume Rambod, Nilofar Pasyar, Ali Mohammad Parviniannasab

**Affiliations:** 1 Community Based Psychiatric Care Research Center, School of Nursing and Midwifery, Shiraz University of Medical Sciences, Shiraz, Iran; 2 Department of Nursing, School of Nursing, Larestan University of Medical Sciences, Larestan, Iran

**Keywords:** Hope, End-stage kidney disease, Hemodialysis, Qualitative study, Content analysis

## Abstract

**Background:**

End Stage Renal Disease (ESRD) patients undergoing hemodialysis are faced with serious problems in their lives. Hope, as a multifaceted factor, plays a critical role in these patients’ lives. Given the multifaceted process of hope, this study aimed to describe hope and identify the challenges, strategies, and outcomes of hope in Iranian ESRD patients undergoing hemodialysis.

**Methods:**

This is a qualitative study using content analysis. The participants were selected using purposive sampling. The data were collected using deep, semi-structured interviews with 14 participants; it continued until reaching data saturation. Graneheim and Lundman content analysis approach was used to analyze the data.

**Results:**

Five main categories and twenty-two subcategories emerged; the categories consisted of (1) Hope described as a particular event to happen, (2) Opportunities and threats to achieve hope, (3) Negative emotions as barriers to achieve hope, (4) Positive coping strategies to achieve hope, and (5) Growth and excellence as the outcomes of hope.

**Conclusions:**

Based on the findings, ESRD patients undergoing hemodialysis described hope as a positive feeling of expectation and desire for a special thing to happen. They faced threats and opportunities to achieve hope, which exposed them to negative emotions as barriers of hope. Thus, they make use of positive coping strategies to achieve hope. Moreover, hope led to growth and excellence. Through awareness of hope, definition and strategies to achieve it, and teaching them, physicians and nurses working in hemodialysis wards can enhance hope in patients.

**Supplementary Information:**

The online version contains supplementary material available at 10.1186/s12882-023-03336-6.

## Background

End Stage Renal Disease (ESRD) is a chronic and life-threatening disease, the prevalence of which is increasing around the world [1, 2]. ESRD is accompanied by disabilities, which reduce the patients’ quality of life [3]. Additionally, this disease can lead to uncertainty, social activity disorders, and disruption of social relationships [4]. Thus, coping strategies should be used in this area. Hope, as a coping strategy, can be used to reduce these limitations [5]. ESRD patients undergoing Hemodialysis (HD) experience better outcomes if they are hopeful [6, 7].

Hope is a process and source of compatibility and adaptation with chronic diseases [8]. It is a multidimensional, universal, and dynamic construct that helps the patient to adjust to the treatment of various diseases. In fact, hope is a cognitive process, in which individuals seek for opportunities, pathways, and probable and specific goals [9, 10]. Hope can help individuals to overcome the darkness resulting from the illness and have the ability to battle through adverse events [8]. It is a complicated process of thoughts, emotions, and functions that change over time and facilitates the achievement of goals and outcomes in future [11]. Evidence has also indicated that hope is related to excellence, acceptance of compatibility [11], depression, mental health, religious beliefs [12], and spirituality [13]. Furthermore, social support and relationship with others have been found to be effective in facilitation and maintenance of hope [14]. Moreover, more hopeful individuals benefit from higher satisfaction with their lives [15].

In a study, it was indicated that family function predicted hope in hemodialysis patients [16]. As mentioned above, most of the studies on hope in hemodialysis patients have been conducted quantitatively [11–13], and these studies could not explain the concept of hope fully with quantitative research. This means that definition of hope is unknown in hemodialysis patients, especially those who lived in Iran as well. Moreover, the challenges and concerns of hemodialysis patients and strategies that these patients use to improve their hope are not well known around the world and in the context of Iran. In addition, with the existing views, it is difficult to explain the hemodialysis patients’ challenges, concerns, and strategies they used to improve their hope, especially in the Middle East countries such as Iran. Therefore, conducting a qualitative research is essential for knowing and explaining this concept and presenting a specific view of it [17]. Qualitative methods help the healthcare workers to describe and understand the concept and obtain in-depth and rich insights [18] about hope. Based on the researchers’ experience in the field of Iranian ESRD and hemodialysis patients, the number of these patients, as a chronic disease, has increased during two decades. Therefore, exploring hope and the factors associated with it using a qualitative approach improves the evidence-base practice in these patients. Thus, this qualitative study aimed to describe hope and identify the challenges, strategies, and outcomes of hope in Iranian ESRD patients undergoing hemodialysis.

## Methods

### Study design

This research was conducted using a qualitative design and content analysis approach. Content analysis focuses on the lived experience, interpretations, and meanings encountered by individuals. Content analysis is usually used in the design of studies the goal of which is to explain a phenomenon. This type of design, unlike the directed method, is often suitable when the existing theories or research literature about the phenomenon under study are limited. In this case, researchers avoid using preconceived categories and instead arrange for the categories to emerge from the data [19].

### Study setting

The study was performed in hemodialysis centers in Nemazi and Shahid Dr. Faghihi hospitals affiliated to Shiraz University of Medical Sciences in Fars province in Southern Iran.

The study participants included 14 males and females over 18 years of age selected through purposive sampling. The inclusion criteria for the patients were speaking and understanding the Persian language, experience of dialysis for at least 6 months, and age over 18 years. Patients in the acute phase of ESRD were excluded from the study.

### Data collection

The study data were collected through semi-structured interviews. Observation and field notes were utilized as well. The total data collection procedure lasted for two months. The interviews were performed in the Persian language by the first author, who was experienced in qualitative studies. The interviews started with the following open questions: “How do you describe hope in your life?”, “How does hemodialysis and renal failure affect your hope?”, “What factors are facilitators and barriers of hope in your life?”, “What circumstances or conditions help you to increase or decrease your hope?”, “What would be the outcomes of achieving hope?”, and “How does achieving hope affect your life?” Then, probing questions were asked, and the patients’ explanations were continued (see supplementary file). These face-to-face interviews were individually conducted at the patients’ bedside during hemodialysis. They lasted for 30–50 min. The interview was done with 14 hemodialysis patients. Two patients were interviewed twice. Therefore, 16 interviews were used for data analysis. All the patients who had inclusion criteria and were invited to the study accepted to participate in the study. Data collection continued until we reached data saturation. All the conducted interviews with the participants were recorded and immediately transcribed verbatim after the end of the interview sessions. In fact, data analysis was done in parallel with data collection by all three researchers. For this purpose, first, the interview of each participant was analyzed and then the next interview was conducted.

### Data analysis

The data were analyzed based on the 5 steps of Graneheim and Lundman’s method [20], including “writing down the entire interview immediately after conducting each interview”, “reading the entire interview text to gain a general understanding of its content”, “determining semantic units and primary codes”, “categorizing similar primary codes into more comprehensive categories”, and “determining latent content in the data”. Thus, semantic units (words and paragraphs that included aspects related to each other) were first formed from the participants’ speeches. Then, the code was extracted from the meaning units. The codes were placed in subcategories, and similar subcategories were placed in one category (as shown in Table 1). MAXQDA 10.0 software was used to organize the data.

**Table 1:** Categories, subcategories, and meaning units of the factors related to hope in the patients under hemodialysis

Main Categories	Subcategories	Meaning unit (primary code)
Hope as particular things to happen	Having positive thoughts	Thinking about positive events, thinking about future and ignoring the renal failure, seeing the future clearly, having good dreams
	Expectation and desire for future things	Being healthy after hemodialysis, coming back home after hemodialysis, being recipient of kidney transplantation, being a healthy person, and having a good life without any disease complication
	Having goal-oriented thoughts, developing strategies to achieve goals, and being motivated to expand effort to achieve goals	Having goals in the life, thinking about buying a house, getting a job in future, waiting for the children go to the university, being successful in the family functions, job, social relationship and life in spite of renal failure and undergoing hemodialysis
	Having family support in future	Having a family that understand, love, care and accept the patients with all of their disease limitations, and living with the husband and children at the same home
Opportunities and threats to achieve hope	Physical challenges	Changes in appearance like turning pale, swelling under the eyes, dark spots on the face, and skin color changes may affect the patients’ hope
	Challenges in social interactions	Dialysis ward environment, presence of other patients, seeing other patients’ problems while communicating with them, restrictions for choosing a place to travel, challenging trips may affect the patients’ hope
	Educational failure	Inability to continue education, physical and mental problems in the classroom may be correlated to hope
	Economic challenges	Losing one’s properties for treating the disease, cost of referral to the hemodialysis center and transplantation, financial problems for following up the treatment process
	Occupational limitations	Job restrictions for patients, obligation for choosing specific jobs, quitting job due to the disease, impatience for finishing works may affect the patients’ hope
	Familial conflicts	Challenges for choosing a spouse, being rejected by the family, inability to fulfill one’s role in the family affect the hemodialysis patients’ hope
	Spirituality	Belief in a supreme power named God as the source of hope, belief in the purposefulness of the world and creation of living things, belief in God as the creator of life, belief in the fact that being thankful leads to blessing in life, and considering the disease as a divine destiny may increase the patients’ hope
Negative emotions as barriers to achieve hope	Depression	Impatience in doing personal and social activities, being intolerant and unmotivated, considering nothing as important in life, being isolated, feeling alone, crying, tendency to die are barriers to achieving hope
	sadness	Being sad due to physical and social limitations are barriers to achieving hope
	uncertainty	Lack of confidence in being ready for transplantation and transplant rejection are barriers to achieving hope
	Anger and hate	Being nervous, getting angry by even simple stimulants, showing aggressive behavior, hatred of dialysis barriers to achieve hope
Positive coping strategies to achieve hope	Positive solution-oriented strategies	Modification of goals, using positive and solution-oriented strategies are to achieving hope
	Staying motivated	Making attempts, listening to music, grooming oneself, banishing negative emotions, taking part in recreational activities to achieve hope
	Positive psychological constructs	Positive thinking, positive expectations, cheering oneself up, comforting oneself, being thankful to achieve hope
	Supportive exchanges	Emotional, informational, instrumental, and spiritual supports to achieve hope
	Connection to transcendence	Talking to God, praying, hope in God, complaining to God to achieve hope
Growth and excellence as outcomes of hope	Improvement of well-being	Optimism, happiness, vitality, being energetic, gaining tranquility, health, building better social relationships with others, promotion of self-efficacy, acceptance of the disease as outcomes of hope
	Finding the meaning of life	Purposefulness in life, finding order in life, following goals in life, being interested in life, recognition of oneself and God, self-care, adherence to treatment, understanding life, love for continuation of life as outcomes of hope

### Rigor

In order to confirm the results and determine the study rigor, we used Lincoln and Guba’s (1985) four criteria, namely credibility, dependability, conformability, and transferability [21]. To gain the data credibility, the researchers listened to the interviews carefully, immersed in the data, selected a variety of patients, and performed the interviews where the participants felt comfortable. To ensure the conformability of the data, the second and third authors of this study reviewed the extracted notes and codes; an external researcher who was familiar with qualitative studies was also employed. To verify the dependability and transferability of the findings, we recorded and reported the steps and process of the research step by step as accurately as possible. In terms of age and gender, the participants were decided to be diverse, which in addition to dependability, also helped the transferability of the findings.

## Results

This study was conducted on 14 hemodialysis patients. There were both male and female patients with the mean age of 32.5 years. Other demographic characteristics of participating patients are summarized in Table 2. During the process of content analysis, five categories, namely hope described as particular events to happen, opportunities and threats to achieve hope, negative emotions as barriers to achieve hope, positive coping strategies to achieve hope, and growth and excellence as outcomes of hope were extracted (Table 1).

**Table 2:** Demographic characteristics of hemodialysis patients who participated in the research (N = 14)

Participant’s No.	Age (year)	Gender	Marital status	Education	Job	Number of years under hemodialysis
P1	35	Female	Married	Academic	Housewife	5
P2	37	Male	Married	Academic	Retired	8
P3	54	Male	Married	Elementary	Self-employed and now, is unemployed	5
P4	25	Male	Single	High school	Employee	4
P5	35	Male	Married	Academic	Employee	6
P6	23	Female	Single	Academic	Housewife	5
P7	25	Male	Single	Elementary	Self-employed	7
P8	25	Female	Married	High school	Housewife	2
P9	35	Female	Single	Academic	Employee	3
P10	41	Male	Married	Academic	Employee	5
P11	35	Male	Married	Secondary	Self-employed	2
P12	26	Male	Single	Elementary	Unemployed	3
P13	33	Female	Single	Academic	Self-employed	7
P14	26	Female	Married	High school	Housewife	1

### Hope described as particular things to happen

The hemodialysis patients described hope as having positive thoughts, expectation and desire for future events; having goal-oriented thoughts; developing strategies to achieve goals; being motivated to expand effort to achieve goals; and having family support in future.

#### Having positive thoughts

Having positive thoughts was reported by two participants (p 11, p 6). *“To me, hope means thinking about positive happenings (p 11), thinking about future, ignoring the renal failure and seeing the future clearly…. I describe hope as having good dreams (p 6)”.*

#### Expectation and desire for future events

Expectation and desire for future events was expressed by participant 3. *“I describe hope as being healthy after hemodialysis, coming back home after hemodialysis, being recipient of kidney transplantation, being a healthy person, and having a good life without any disease complication (p 3)”.*

#### Having goal-oriented thoughts, developing strategies to achieve goals, and being motivated to expand effort to achieve goals

The patients described hope as having goal-oriented thoughts, developing strategies to achieve goals, and being motivated to expand efforts to achieve goals. In this regard, participants 4, 6, 5, 14 said *“To me hope means having goals in the life (p 4)”. “I think about buying a house and getting a job in future in spite of having renal failure. I wait until my children go to the university, and get married (p 14)”. “I think hope is being successful in my family functions, job, social relationship and life in spite of renal failure and undergoing hemodialysis. In other words, I describe hope as being successful in education and university, and renal failure and hemodialysis do not affect my life (p 6)”.*

#### Having family support in future

Having family support in future was described by the hemodialysis patients as the meaning of hope. *“To me, hope means having a family that understand, love, care, and accept me with all my disease limitations (p 11). I express hope as living with my husband and children at the same home (p 13).*

### Opportunities and threats to achieve hope

Perception of opportunities and threats to achieve hope was extracted from the participants’ experiences. On one hand, the patients faced such threats as physical challenges, challenges in social interactions, educational failure, economic challenges, occupational restrictions, and familial conflicts to achieve hope. On the other hand, they pointed to the negative and positive effects of the disease and hemodialysis. Furthermore, spirituality alongside the positive impacts of treatment provided a great opportunity for facing the threats.

#### Physical challenges

The hemodialysis patients have physical challenges including skin and nail change, fatigue, weakness, bone pain, difficulty sleeping, muscle cramps, nausea, drowsiness, etc. that affect the patients’ hope. Patients have facial changes such as paleness, swelling under the eyes, spots on the face, changes in the appearance color, and, in other words, a sickly face. In addition, the smell of urea can come out of their mouths. Sometimes, the accumulation of toxic substances in the body causes nausea and anorexia in the patient. In this regard, two participants (p 14, p1) expressed *“When my kidney failed, I turned pale; there was swelling and edema under my eyes down to my cheeks, and I had pain all over my body. From then on, I haven’t been able to sleep or sit; I suffer from shortness of breath; these may affect my hope”* … (p 14); “*We undergo dialysis 3 times a week. This causes our physical strength to decrease. I became very weak and do not drink water and liquids as much as we want. I feel nauseous and I have no appetite*…,* I think these impact my hope*” (p 1).

#### Challenges in social interactions

Hemodialysis clients are limited in their social activities due to dependence on hemodialysis machines 2 or 3 times a week. Moreover, because of ESRD, they have no physical energy, and are not interested in communicating with others and participating in social activities. Based on the patients’ reports, these challenges in social interactions my influence the patients’ hope. Statements of the participants in this regard are presented below:

*“We have restrictions on commuting; for example, there must be a dialysis center in the place we intend to visit, so that it is close to us in terms of location*…” *(p 10); “As much as we want, we don’t have free time to be with friends and relatives. It means that due to our condition and dialysis, we cannot be together andachieve hope*; *that’s why, our communication has decreased…” (p2)*.

#### Educational failure

Inability to continue their education and studies and physical and mental problems in the classroom are threats to the lives of ESRD patients undergoing hemodialysis and affect the patients’ hope. They stated:

“*I was accepted in the university. I was not able to study. The disease ruined all my thoughts; I had no motivation and did not have patience for this work. I had no energy…, so how I can be hopeful?”* (p 4); *“I really could not go to class. You know, I had dialysis in the morning three days a week. I had to go to class in the evening, and I really could not sit in class. Every time I went, I was so sleepy and dull that I can’t tell…” (p13)*.

#### Economic challenges

The cost of going to and from the dialysis center and the existence of financial problems to follow the treatment processes are some of the economic challenges. Two participants (p 8, p 3) stated:

*I sold my house and shop. In these 4 years that I have been ESRD, I spent all my family money on treatment and doctor’s fees …. These led to loss of hope during these years.” (p 8); “I was at work for 12 years when I was transplanted, but when I got dialysis, I said I can’t come, and I am unemployed now….” (p 3)*.

#### Occupational restrictions

Being limited in work for patients, having to engage in certain tasks, being unable to work, giving up work at the onset of illness, and being impatient to finish work threatened the hemodialysis patients. Two participants’ expressions (p 12, p 11) are as follows:

*“We cannot choose any job. Many of us do not go to work after we get dialysis…” (p12); “We are really having trouble finding a job. We are all involved in the hospital and tests, but our conditions do not allow us to find a job…” (p 11)*.

#### Familial conflicts

These patients have difficulty choosing a spouse. They face family rejection, family disputes, and inability to fulfill family roles. These may affect the patients’ hope. In this regard, one of the participants said: “*Since my illness was diagnosed, my wife has gradually moved away from me; we don’t have the same interest and love as before… this bothers me…you know, my family is my hope” (p 10);* “*I have two children. Now that I go to dialysis three days a week, I really can’t see my children…” (p 1)*.

#### Spirituality

Five participants (p 5, p 7, p 9, p 11, p 13) believed in a supreme power called God as the source of hope. They believed in the purposefulness of creation of living things, were thankful to God, and believed that the disease had come from God, and God would help them. In this regard, two participants (p 5, p 9) mentioned:

*“God wanted me to become sick. I know that the disease has come from God. God wanted to tell me to be careful. I think the disease is an examination sent by God…” (p 5); “I know that every problem and pain that happen to me, are from God. I say, God, I will accept whatever you give me. I say, God, you are right. If you have faith in God, you will find faith in yourself, and you will find purpose and hope in your life…” (p 9)*.

### Negative emotions as barriers to achieve hope

Depression, sadness, uncertainty, anger, and hate were the main concerns of the patients that were barriers to achieve hope. These patients were isolated, felt lonely, cried, and talked about tendency towards death. They were also unhappy with their physical and social limitations. Moreover, they were not certain about future and disease complications. Some patients were nervous, lost their temper easily, and showed aggressive behaviors. They said that these negative emotions were the barriers of hope. Besides, some of them hated the treatment process.

#### Depression

These patients showed signs and symptoms of depression. They felt tired and energy less. They lost their interest and pleasure in most or all-normal activities. The patients also reported tearfulness and hopelessness. They felt worthless and had frequent thoughts of death.

Two participants (p 13, p 10) stated:

*“The disease ruined my motivation and my boredom. I got tired of the disease, treatment, and its side effects…” (p 13); “The disease had a bad effect on my mood. I will not live anymore. There is no hope for me to survive. I should get a kidney and be transplanted. . I do not want to undergo dialysis anymore. In the end, I am seeing death…” (p 10)*.

#### Sadness

Patients were upset about starting dialysis to treat the disease, not being with their family members during the week, and not being able to fulfill some of their tasks and roles for their children.

A participant said in this regard:

“*On days of dialysis, I can’t go to a party with my family. Well, they certainly like us to be together, but I cannot. I get upset and sad…you know, it leads to loss of my hope” (p 12)*.

#### Uncertainty

Seven participants mentioned their uncertainty about whether they can get a transplant, as well as the complications associated with a transplant. One of them said:

*“I am undergoing dialysis. I am also a transplant candidate. I see people who were transplanted, and it did not work… Now they are undergoing dialysis. I tell myself that this problem should not happen to me. This uncertainty is frustrating…and leads to loss of my hope” (P1)*.

#### Anger and hate

Patients have become nervous since the onset of the disease, very quickly become frantic due to a simple shock, and show aggressive behavior. In addition, hatred towards dialysis and the equipment of the dialysis department was evident as another negative emotional reaction. One of them said:

*“I hate dialysis and the equipment of the dialysis department. When I want to go for dialysis, I get very upset that day…” (p 3)*.

### Positive coping strategies to achieve hope

Positive coping strategies refer to the behaviors shown by eight participants for solving their concerns in the process of hope. In fact, the participants used positive and solution-oriented strategies, stayed motivated, utilized positive psychological constructs, had supportive exchanges, and were connected to transcendence to cope with negative emotions and achieve hope.

#### Using positive and solution-oriented strategies

The patients with ESRD tried to have purpose in their lives, modify their goals, and make use of positive and solution-oriented strategies to cope with their negative emotions and are hopeful. In this regard, one of the participants stated:

*“I am counting the days to reach my goal. When a person has a goal in his/her life, he/she becomes hopeful. When there is hope in life, life becomes orderly…” (p 2)*.

#### Staying motivated

The patients with ESRD made genuine attempts to stay motivated by using constructive approaches such as trying, listening to music, grooming themselves, avoiding negative memories, and taking part in recreational activities to overcome negative emotions and achieving hope. In this regard, two participants stated:

*“When I think about my kidney, to avoid getting depressed, I stand in front of a mirror. I comb my hair, change my clothes, and wear perfume…”* (p 7); “*When I go to a recreational place with my family or friends, I get a lot of energy… my motivation and hope to continue life increase*…” (p 9).

#### Using positive psychological constructs

To cope with negative emotions and achieve hope, the participants used positive psychological constructs including positive thinking, having positive expectations, cheering themselves up, comforting themselves, and being thankful. Statements of the participants in this regard are presented below:

*“See! I always try to see the glass half full. I tell myself that the disease is there, and I have to think positively about it…”* (p 6); “I *compare myself with other diseases such as cancer and thalassemia. Thalassemia patients have many appearance and physical problems. I console myself and say, thank God, there is dialysis. And we hope…” (p 14)*.

#### Supportive exchanges

Holistic support was found to be a strong strategy for coping with the disease and achieving hope. This included emotional, informational, instrumental, and spiritual support. Two patients’ expressions (p 9, p11) are as follows:

*“In this world, you can only count on your family…Your family gives you hope…Your family always stays by your side…”*(p 9); “*Whenever I have a question, I ask the hemodialysis center; there is also a counseling room here… I ask a lot of questions, and this helps me to reduce my problems* …” (P 11).

#### Connection to transcendence

Connection to transcendence was another strategy used by the patients to achieve hope. This was done through talking about God, praying, having hope in God, and complaining to God. In this regard, three participants (p 9, p 5, p 10) stated:

*“I asked God to heal me. I easily talk to God…”* (p 9); “*I am a person who prays and even attend religious ceremonies. Well, this gives me peace. You know that God always cares about His servants… With this disease, my relationship with God has increased*…” *(p 5); “Now that I have this disease, I am closer to God… There is not a day that I do not wake up and call the name of God… “(p 10)*.

### Growth and excellence as outcomes of hope

Using the above-mentioned positive coping strategies to improve hope and eliminate negative emotions led to growth and excellence. In other words, hope resulted in the improvement of well-being and finding the meaning in life.

#### Improvement of well-being

Utilization of positive coping strategies led to optimism, happiness, vitality, full energy, tranquility, health, and better social relationships with others, promotion of self-efficacy, and disease acceptance. In other words, using these strategies improved the patients’ well-being.

In this regard, two participants (p 14, p 7) stated:

*“Hope gives you positive energy. It gives you a feeling of happiness and increases your energy. When you are hopeful, you laugh; you think about good things; you have good dreams in your mind, positive dreams for future, and good feeling; and you feel relaxed…”* (p *14);* “*Hope keeps you physically and mentally healthy. It reduces the disease symptoms and pain. Hope leads to living, earning money, working, everything…I’m sure that I’ll be successful in my life and in my job in future…” (p 7)*.

#### Finding meaning in life

Utilization of positive coping strategies helped the patients with ESRD find meaning in their lives. In other words, they became purposeful, found order in their lives, and pursued their goals. They were interested in life, recognized themselves, achieved faith in God, understood life, and were able to care for themselves. In this regard, two participants (p 1, p 7) stated:

“*Maybe, I didn’t think about life very deeply before I got sick…but now that I’m sick and I’m having a hard time, I appreciate life more. I just understand how sweet life can be despite the hardships. Life seems to have a different meaning to me than when I was healthy… “ (p 1)*; “I *know that a person should be comfortable and hopeful for himself… these are necessary to endure problems; I don’t know that maybe this disease will bother us more than what it is now… but I am hopeful about the future and have a happy ending. It makes life sweeter for us…”* (p 7).

## Discussion

The results of this study indicate that hope in hemodialysis patients means having positive thoughts, expectation and desire for future events and goal-oriented thoughts; developing strategies to achieve goals; being motivated to expand effort to achieve goals; and having family support in future. In a qualitative study, hope was reported that as “lively, active, and, above all, a personal choice”. Hope was the desired possibility; however, it needs an effort to be achieved [22].

The findings of the present study showed that threats to achieve hope in hemodialysis patients consisted of physical challenges, challenges in social interactions, educational failure, economic challenges, occupational restrictions, and familial conflicts. Although no qualitative study have reported the factors that affect hope in hemodialysis patients, a qualitative study showed that mental well-being and depression were associated to hope in patients who have undergone dialysis [23]. The results of other studies indicate that hemodialysis patients face many physical, social, and financial complications due to the nature of their illness and treatment [24, 25]. In the qualitative studies conducted in Iran, fatigue, physical disability, social limitations and financial and employment problems were some of the challenges based on the experiences of hemodialysis patients. It seems that the continuation of the disease and treatment with dialysis and changes in the lifestyle, such as financial problems, unemployment, and changes in family duties and roles may affect the hemodialysis patients’ hope.

Despite these threats, the patients studied revealed opportunities like spirituality, which were effective in the process of hope. Five participants believed in a supreme power called God as the source of hope. In general, Muslim patients consider diseases as the divine destiny that remove their sins. In line with the current study results, other studies demonstrated that spiritual experiences increased hope and had a positive effect on the reduction of stress [7, 26, 27]. Another study reported that hope was associated with spirituality in patients with chronic kidney disease [9]. It seems that belief in God in patients makes them see God’s hand in all matters; as a result, they tolerate discomforts of life better, protect themselves against accidents, and get more patient.

The findings of this study showed that the patients who were undergoing hemodialysis encountered negative emotions including depression, sadness, uncertainty, anger, and hate as barriers to achieve hope. A qualitative study on end-of-life patients reported that “limitations imposed by illness, feelings of anguish and helplessness and poor communication with clinicians” were barriers of hope [22]. A quantitative study indicated that in chronic diseases hope was negatively associated with depression and anxiety. Moreover, hope can change the relationship between uncertainty and depression and anxiety symptoms [28]. Review of the literature also revealed that hemodialysis patients experienced stress in their lives [7, 26, 29, 30]. In general, the physical and social limitations of hemodialysis patients, along with fear, anxiety, uncertainty, and limitations of recreational activities and dialysis treatment make depression inevitable [31, 32]. Fear of future and uncertainty seem to be due to the fluctuating conditions that patients experience during treatment as well as complications of the disease that may affect the patients’ hope.

Positive coping strategies were another finding of the study to achieve hope. Hemodialysis patients used positive solution-oriented strategies, positive psychological constructs, and supportive exchanges; connection to transcendence; and motivation to achieve hope. In line with our study, a qualitative research on end-of-life patients reported that “supportive others, positive thinking and sense of humor, connection with nature, faith in religion and science, and a sense of compassion with others and altruism” were facilitators of hope [22]. The results of other studies indicate that there is a positive relationship between hope and optimism and positive coping style [27, 33]. As a result, people who have a positive coping style are more likely to understand cognitive optimism and make positive behavioral efforts to cope with illness, which is useful in creating positive psychological concepts such as hope [34].

Supportive exchanges were also the strategies used for overcoming negative emotions and developing hope in hemodialysis patients. In various studies, the role of social support and its effect on improving hope have been proven, which is consistent with the present study [13, 14, 35]. Moreover, researchers have reported that the hemodialysis patients who benefit from higher social supports are more hopeful [12]. It was also shown that if a person receives support from a network and a peer, s/he can cope with difficult situations of the illness, which in turn raises the level of hope [14].

In the current study, growth and excellence were the other prominent strategies related to hope in patients under hemodialysis. In other words, hope resulted in the promotion of the patients’ well-being, which helped them find meaning in their lives. In various studies, in line with the current research, the use of positive coping strategies by patients with ESRD under hemodialysis leads to happiness, vitality, disease acceptance, and well-being [36, 37]. Another study reported that hope was associated to better quality of life in hemodialysis patients [7]. When patients are hopeful, they can benefit from their energy to rebuild their health and well-being [38]. The results of another study on hope among chronic patients indicated that hope led to the improvement of health, facilitation of compatibility, promotion of life quality, and improvement of self-esteem [39]. In the present study, using positive coping strategies in response to negative emotions resulted in the meaningfulness of life. In other words, the patients became purposeful and found order in their lives. They pursued their goals and made attempts to reach their ideal lives. Evidence has shown that hope is the key to spiritual well-being and encourages the individuals to move and act in their lives [27, 40].

### Limitation

We used qualitative content analysis to examine the patients’ experiences of hope. Using a design such as phenomenology can help to better understand the patients’ lived experience of this concept. In addition, the lack of similar studies inside the country limited the possibility of comparing the findings with local studies. For this reason, repeating similar studies can help clarify the concept and its effective factors.

### Strength

To the best of our knowledge, this is the first study which aimed to describe hope and identify the challenges, concerns, strategies, and outcomes associated with hope in Iranian ESRD patients undergoing hemodialysis. An in-depth interview with maximum diversity and the use of purposeful sampling was the main strength of this study. The qualitative approach can also help us to describe hope and deeply understand the factors related to hope in patients with ESRD who are under hemodialysis, which can be fundamental for designing nursing interventions.

## Conclusion

The results of this study indicate that hope in hemodialysis patients means having positive thoughts, expectation and desire for future events, and goal-oriented thoughts; developing strategies to achieve goals; being motivated to expand effort to achieve goals; and having family support in future. The study findings indicated that the patients with ESRD who were undergoing hemodialysis were faced with threats and opportunities to achieve hope. These caused them to develop negative emotions as barriers to achieve hope. In response to these concerns, they made use of positive and solution-oriented strategies, stayed motivated, used positive psychological constructs, had supportive exchanges, and resorted to transcendence to achieve hope. Being hopeful eventually led to growth and excellence. Therefore, there is a need for educational programs to be held with a focus on the use of active and constructive coping styles to increase hope. Thus, healthcare providers should support the patients to achieve coping strategies and in turn create hope and motivation to cope with stress and emphasize active coping in the daily life of these patients; also, the supporting role of the family is emphasized to remain hopeful to effectively deal with the disease and its treatment.

### Electronic supplementary material

Below is the link to the electronic supplementary material.

Supplementary Material 1

## Data Availability

The datasets generated and/or analyzed during the current study are not publicly available due to the necessity to ensure the participants’ confidentiality policies and laws of the country but are available from the corresponding author on reasonable request.

## Abbreviations

ESRD: End-Stage Renal Disease
HD: Hemodialysis
