# Supplementary material for: A qualitative study on hope in iranian end stage renal disease patients undergoing hemodialysis
Source: BMC Nephrol. 2023 Sep 22;24:281. doi: 10.1186/s12882-023-03336-6 (PMC10517523; doi:10.1186/s12882-023-03336-6)
Supplement: Supplementary file 1 — Supplementary Material 1 [file 12882_2023_3336_MOESM1_ESM.docx]

Table1. Hope interview questions

| **Main questions** | **Guiding questions** |
| --- | --- |
| 1. How do you describe hope in your life? 2. How does hemodialysis and renal failure affect your hope? 3. What factors are facilitators and barriers of hope in your life?”, “What circumstances or conditions help you to increase or decrease your hope? 4. What would be the outcomes of achieving hope? 5. How does achieving hope affect your life? | 1. Can you explain more? 2. What do you mean, why and how? 3. Can you give an example? |
